# Mind Everywhere: A Framework for Conceptualizing Goal-Directedness in Biology and Other Domains—Part Two

**DOI:** 10.1007/s13752-025-00524-5

**Published:** 2026-02-25

**Authors:** Michael Levin, David B. Resnik

**Affiliations:** 1 Allen Discovery Center, Tufts University, Medford, MA, USA; 2 Wyss Institute for Bioinspired Engineering, Harvard University, Boston, MA, USA; 3 National Institute of Environmental Health Ethics, National Institutes of Health, North Carolina, USA

**Keywords:** · Agency · Biomedicine · Bioengineering · Developmental biology · Diverse intelligence · Philosophy · Teleology

## Abstract

What makes a system—evolved, engineered, or hybrid—describable by teleological and mentalistic terms such as intelligent, goal-directed, cognitive, and intentional? In this two-part article, we review classical thought on teleology in the life sciences and defend a new approach to goal-directedness that stems from an emerging field—diverse intelligence. This field seeks to characterize what all active agents, regardless of their composition or provenance, have in common. Our approach emphasizes: (1) empirical testability (not philosophical commitments to linguistic categories), (2) fecundity in discovery of new capabilities (not just reductive mechanistic explanations of results after they are made, but worldviews that facilitate and enable novel research), (3) operationalization of terminology by reference to conceptual and empirical toolkits shown to be effective for a given system (cognitive and teleological claims are really hypotheses of optimal interaction protocols), and (4) continuity of human goal-directedness with our unicellular origins (which implies a need for models of scaling of cognition). In Part One, we reviewed historical and contemporary debates about teleology in biology and described some ground-breaking experimental results from morphogenesis, synthetic biology, and other areas of research involving the coordination of cellular behavior by means of bioelectric networks. In Part Two, we argue that current approaches to goal-directedness in biology cannot accommodate these results in a manner that helps us better understand how these systems work and fruitfully guides experimental work and practical applications in these areas of research. What is needed, therefore, is an approach to teleology in biology, informed by mentalistic concepts, such as intelligence, cognition, and intentionality. We describe and explain our mentalistic approach in greater depth, show how it can be applied to specific cases, and address some important objections to our view. By abandoning the reductionistic aversion to mentalistic concepts in biology in favor of principled and testable frameworks for understanding diverse minds embodied in the physical world, the plasticity and problem-solving competency of the agential material of life can provide fodder for advances in philosophical thought, as well as for biomedical and bioengineering applications.

## The Need for a New Approach to Goal-Directedness in Biology

This is Part Two of the article in which we defend a new approach to goal-directedness in biology called *Technological Approach to Mind Everywhere (TAME)*. In Part One, we reviewed historical and contemporary debates about teleology in biology and described some ground-breaking experimental results from morphogenesis, synthetic biology, and other areas of research involving the coordination of cellular behavior by means of bioelectric networks. In this article, we argue that current approaches, which tend to minimize the emphasis on goal-directedness in biology (and thus suppress the use of powerful tools from behavioral science) cannot account for these experimental results in a manner that helps us better understand how to control living systems toward new large-scale functions and fruitfully guide novel experimental work and practical applications in these areas of research. What is needed, therefore, is an approach to teleology in biology informed by well-defined, rigorous, theory-backed mentalistic concepts, such as intelligence, cognition, and intentionality (and their attendant conceptual tools from neuroscience). In this part, we will also describe and explain our mentalistic approach in greater depth, show how it can be applied to specific cases, and address some important objections to our view. By abandoning the reductionistic aversion to mentalistic concepts in biology in favor of principled and testable frameworks for understanding diverse minds embodied in the physical world, the plasticity and problem-solving competency of the agential material of life can provide fodder for advances in philosophical thought, as well as for biomedical and bioengineering applications.

## Goal-Directed Models as Contrast and Complement to Mechanistic Frameworks: Optimizing Experimental Fecundity

Current approaches to goal-directedness in biology focus on the rules of chemical interactions within protein pathways, dynamical systems, and emergence via complexity arising from iteration of low-level rules in large numbers of parallel cells or networks (agent-based modeling, cellular automata, and so on). These approaches are dominated by discussions of signaling pathways, mechanical networks of forces, and other mechanistic metaphors, but also increasingly include gradients of attractive or repulsive stimuli, measurement, precision and error, memory, decision-making across various landscapes (Bugaj et al. 2017; Eritano et al. 2020; Houchmandzadeh et al. 2002; Moris et al. 2016; Rabinowitz et al. 2017; Spirov and Holloway 2002; Tamari and Barkai 2012). While very few approaches explicitly emphasize the agency of the various biological components and use appropriate terms from behavior science to modulate them (Pezzulo and Levin 2015, 2016), it is clear that even the current teleophobic paradigm is not as far from the right side of the spectrum of persuadability (Part One, Fig. 1b) as one might think. What are the arguments for pushing further in that direction? Are not mechanistic molecular biology and genetics sufficient? And if they are, might not one reduce further? Would models formulated in terms of quantum foam help design medical therapeutics or predict form and function in evolutionary outcomes?

It is important to state clearly what we are *not* claiming. (1) We are not claiming that teleological terms should simply be added wherever possible for their own sake. Ours is a pragmatist claim: the applicability of mentalistic metaphors, at any level, needs to be backed up by the experimental utility of doing so. They can be neither ruled out nor claimed in any particular case without experimental support. (2) We are also not using the very few examples covered above as the entire evidence for their utility. Those are representative examples, but there is a huge field of work in diverse intelligence, evolutionary ecology, developmental biology, synthetic bioengineering, and so on, in which many more examples have been and increasingly are found. These ideas do not stand or fall on the basis of any one experimental approach or dataset—they represent a philosophical perspective on the mechanistic and algorithmic continuity revealed by the evolutionary origins of the one uncontroversial example of goal-directed physical beings: brainy organisms. Bioelectricity, while a very convenient system in which to demonstrate the concepts we’re dealing with (because of its symmetry with neural processes), has no monopoly on these properties. Ultimately, what we are describing is not the story of bioelectricity but the story of a continuum of cognitive capacities emerging in a wide range of embodiments (Levin 2022). And, (3) we are not claiming that any results in this field will be incompatible with the facts of molecular biology or other underlying mechanisms.

This last part is crucial: it is guaranteed that, whatever remarkable example of goal-directed activity one studies, whether in humans, tissues, or ant colonies, there will be some physical mechanism underlying it. It will never be magic, and if one wants to look at the lowest level, one will always find chemistry (and quantum mechanics underneath that). What this fact does not warrant is an assumption that taking this level of description is optimal. First, because it is now known that some systems really do have causally potent higher levels of organization (Albantakis et al. 2017; Cliff et al. 2017; Hoel and Levin 2020; Hoel 2017, 2018; Hoel et al. 2013b, 2016; Lizier 2014; Lizier et al. 2011; Wang et al. 2012; Wibral et al. 2014) that do the work that their parts do not do. Second, telling stories consistent with molecular biology *after* something has been discovered is not the same as having a generative paradigm that leads to those discoveries in the first place. One feature that the examples in Part One (Figs. 2, 3 and 4) have in common is that, while all of these capabilities are consistent with the known properties of genes, proteins, and so on that implement them, knowing those properties was not sufficient to discover them. They were found by making and testing hypotheses driven by the specific research program of extending mentalistic toolkits to other embodiments and to novel problem spaces (as guided by evolutionary and developmental continuity).

Thus, we argue that the common preference to give explanations at the lowest level of description should be moderated or replaced by the necessity of giving explanations that not only work backwards and are consistent with what was found but also enable new findings in the future. We favor a pluralism in which different types of explanations can coexist, but importantly, there are clear criteria for adjudicating among them. Fecundity is one, but it is only recognized on the scale of decades. Another, more immediate sign is *efficiency* of control: whereas the mainstream approach might call for careful control of a myriad of cells or genes to produce an eye (Pai et al. 2012) or regenerate a vertebrate leg over a year and a half by a stimulus provided over the first 24 h (Murugan et al. 2022), an approach focused on prompting the native problem-solving intelligence of cells does it with a very low information content trigger. The ratio of information and effort invested versus the results obtained by the engineer or worker in regenerative medicine is a reliable indicator of whether one has identified an optimal level of interaction with a complex system. Knowing how much needs to be micromanaged and how much ingenuity, memory, autonomy, and so on can be expected from a system is the tractable research program of placing cells, tissues, and so on at the right place on the continuum (in turn, determined by whether you get better payoff from genomic rewiring or the use of tools like training paradigms, psychoactive compounds, communication protocols, and so on, or both).

Regenerative medicine and bioengineering are areas in which the practical consequences of different philosophical frameworks play out (Lagasse and Levin 2023, 2013b ; Levin 2024a). The conventional, non-mentalistic, approach to causation in biology, with emphasis on complex but mechanical (indirect, but not cognitive) linkage between genotype and phenotype, does not facilitate discoveries regarding the wide plasticity and reprogrammability of life. The causal role approach, which construes goal-directedness as cause/effect relationships in hierarchically organized biological systems, does not suffice either. To detect, and functionally exploit goal-directedness, one needs an account of how information is represented in the network and how interventions at the level of the network can affect that information (Levin 2022), but the casual role approach does not address this issue, because it treats biological systems as elaborately organized mechanisms, like clocks, rather than as information-processing systems which can implement different degrees of sophisticated, valenced, decision-making. A standard computationalist approach is likewise often insufficient because biological systems operate with a polycomputing architecture where the mapping of physical events onto salience and meaning is performed by different observers at all scales within the body, all of whom interpret it differently (Bongard and Levin 2023) based on their prior memories, goals, and cognitive capacities.

A proponent of the causal role theory could reply that the information contained in bioelectric networks could, in theory, be explained in terms of the electrical connections among cells in the network. But the task of explaining problem-solving capacities of bioelectric or biochemical network connections among cells would be equivalent to claiming that computer algorithms do not really exist and only Maxwell’s equations should be used on the electron flows to understand and make use of computers (Auletta et al. 2008; Ellis 2008, 2009, 2012; Ellis and Bloch 2011). For the same reason, no one has been able to show (yet) how to reduce information contained in highly complex bioelectric networks, such as brains, to physics or chemistry (Chalmers 1997; Dennett 1989; Penrose 1989; Searle 2005). In our critique of the causal role view, we do not assume dualism, vitalism, or any other exotic metaphysics. We are only claiming that to understand and fully exploit the remarkable capabilities of biological systems in novel circumstances, one must ascend above the level of physics and chemistry and consider how information in the networks is represented and processed (Levin 2022).

Fields (Grodstein et al. 2023; Wartlick et al. 2009; Wolpert 1969) are a helpful contribution to the project of explaining goal-directedness in biology. An epistemically sophisticated concept of a field (that is, a field that contains information) can play a useful role in explaining the behavior of complex biological or mechanical systems. But like the local microenvironmental features that cell biologists study, on their own, fields do not suffice—it is still critical to understand how they are interpreted by the agential medium of life, which makes decisions in light of past experience and preferred future states. Also, causal role theories can be useful in helping scientists to understand the relationship between different levels of organization in biological or mechanical systems. Borrowing a distinction from Mayr (1961, 1992) (see also Laland et al. 2011), we can say that history provides distal explanations of goal-directedness in mechanisms such as bioelectric networks. Still, the information contained in these networks and the molecular machinery that executes it provides proximate explanations.

The concept of biological inherency, emphasized by Newman and colleagues, is also a useful contribution to explain and predict certain aspects of goal-directedness in biology (Newman 2022, 2023, 2024; Newman et al. 2025). For example, to explain and predict cleavage, blastulation, gastrulation, and differentiation in a developing embryo, it is useful to know that cells have the inherent ability (and indeed, an autonomous drive) to form certain structures that arise during these processes (such as a blastula, gastrula, appendage, and so on). However, to explain specific instances of not only normal development, but also its remarkable adjustments to surprises in both environmental features and internal components (Hartl and Levin 2025), it is essential to develop frameworks that show exactly how the cell collective-as-agent navigates the space of possible affordances that can be used to reach target morphology (or improvise a novel one, such as in the case of synthetic morphology (Gumuskaya et al. 2025). We suggest that a natural complement to the ideas of Newman et al. is to examine the use of well-established tools of cybernetics and behavioral/cognitive science, as formalisms for investigating (and building) multiscale, agential materials that solve problems and discover novel spaces in which to thrive. Historical approaches can also play an important role in helping biologists understand goal-directedness. Historical approaches might lead biologists to hypothesize that bioelectric networks have played an important role in macroevolution by providing organisms with tools for enhancing variation, adaptation, speciation, reproduction, and survival (Levin 2014, 2023a, 2023b, 2023d). For example, bioelectric networks probably contributed to the success of the first multicellular organisms (about 600 million years ago) by facilitating the coordination and control of individual cells to achieve group goals (Levin 2023a, 2023d). Evidence for this hypothesis comes from experiments demonstrating that bacteria growing in biofilms utilize bioelectric networks to regulate their behavior (Levin 2023a). Fossil records indicate that organisms started forming biofilms shortly after life originated, about 3.8 billion years ago (Römling 2023). So, it is likely that bioelectric networks have evolved over time and become more sophisticated as life has increased in complexity (Levin 2023a). The chemical and electrical activity in nerve tissue, brains, and other components of the nervous system is a natural extension of preexisting cellular networking capacities (Levin 2023a). While biologists have long understood that even very simple nervous systems can store and process information, it is important to recognize that this ability is no different from what cells have been able to do for eons. Recent computational modeling efforts have shown how the process of evolution is accelerated and made more robust when the material, over which evolution functions, has active, computational, and agential properties in the layer between genotype and phenotype (Hartl et al. 2024; Shreesha and Levin 2023).

If leading approaches to goal-directedness cannot adequately explain how information guides cells in bioelectric networks, what kind of approach is up to this task? We believe that what is required is an approach that takes seriously the notion that biological systems can receive, store, process, and utilize information related to goals, and employ a variety of means to attain them. That is, an approach that characterizes biological systems as goal-directed agents that have at least a rudimentary form of intelligence. In the next three sections of our article, we describe our mentalistic approach—known as Technological Approach to Mind Everywhere (TAME) (Levin 2022) in greater depth, show how it can be applied to specific cases, and consider some potential objections to it.

## Technological Approach to Mind Everywhere

Because TAME signifies a partial return to the widely disfavored (and many would say, discredited), mentalistic approach to goal-directedness in biology, we need to clarify what we mean by “intelligent,” “goal-seeking,” “agent,” and other key concepts, in order to avert unfair critiques or misunderstandings of our view. The elements of our approach are as follows:

1. We follow William James (1890) in defining intelligence as the capacity to achieve a goal by different means. Intelligence comes in degrees, depending on the capacities of the agent (Levin and Dennett 2020). The highest forms of intelligence involve consciousness, perception, problem-solving, and metacognition, but lower forms need only involve persistence and plasticity with respect to goal-promotion. Thus, when we describe groups of cells as being goal-directed, we are not claiming that cells are goal-directed or intelligent in the way that human beings are, only that they have certain capacities related to achieving goals.
2. By an agent, we mean a system with the capacity to promote its goals. An agent could include a cell, organism, person, or social group (Schlosser 2019). Agents are always composed of parts, and collections of components are agents to the extent that the parts are sufficiently integrated and well-functioning to achieve the agent’s goals—ones that are of a scale, or operate in a problem space, that differs from those of the parts. An agent need not be conscious or even alive to any significant degree; for example, agents could include viruses, computer programs, corporations, or robots. A significant agent executes a perceptual control loop, in which it takes measurements, makes comparisons to a setpoint, and tries to minimize error with respect to its setpoint and prior expectations. Agents are demarcated by a cognitive light cone determined by the spatiotemporal size of the goals they can pursue (the area of spacetime in which states can motivate corrective action and stress). Higher-level agents sense, act, and make models of the world around them (and themselves). Agents can be described at multiple scales, including the microscopic scale that emphasizes determinism and backward-looking explanations for events. Use of the word “agent” refers to a frame that emphasizes: (A) third-person recognition of a system with competency to navigate problem spaces. Low-agency tools (for example, the tools of physics) are not good at detecting agency (this is why they see chemistry, not minds). Agency is best detected by another agent’s cognitive processes (that is, by external observers such as scientists, parasites, and conspecifics), via perturbational experiments. Detection of agency is fallible, observer-dependent (not objective), and empirical. (B) Second-person perspective—attempts to control other agents in a communication/instruction mode—tools along the Continuum of Persuadability (Levin 2022) (again, dependent on the skill of the observer to deploy the correct tools to exert control and hack the agent to desired outcomes).
3. A goal is an outcome, event, or situation promoted by an agent, such as energy acquisition, information maximization, a specific shape, a specific physiological state, nutrition, health, survival, reproduction, a temperature setting, shelter, territory, companionship, or profit. Goals can be formulated in very concrete spaces (for example, the goal of drinking water from a stream) or in abstract ones (the goal of living a good life). Different types of agents have different types of goals, and agents expend energy to reach specific states (goals) in diverse problem spaces with different degrees of ingenuity. A goal exists whenever the tools of goal-based frameworks (for example, cybernetics, control theory, behavioral science) provide good traction on the system (prediction and control). This does not need to be “purpose” (high-level goals where an agent has the metacognition to think about having goals and what they might be). The word “goal” is useful to indicate situations where an engineer, conspecific, parasite, or other agent does not need to micromanage some aspects of the system because they can offload that onto the system itself (when it has some ability to pursue goals without needing to be micromanaged at each step).
4. An agent promotes (or pursues) a goal by taking action(s) to achieve it, regardless of whether the agent obtains the goal or even can obtain it. For example, we would say that a developing human embryo has the goal of forming a human child even if the embryo spontaneously aborts. We would also say that a human political organization has the goal of promoting a just society, even if this goal cannot be fully achieved.
5. Goal-promoting comes in degrees, depending on the agent’s capacities. The most advanced forms of goal-promoting involve intentional (conscious, deliberate) action, but less advanced forms need only involve persistence and plasticity with respect to a goal. For example, we would say that a thermostat at home is an agent that takes action to regulate the temperature in the house by controlling the heating, ventilation, and air conditioning system in response to temperature readings provided by a sensor.
6. The degree to which an agent is intelligent corresponds (roughly) to its possession of some of the following proposed^1^ characteristics associated with intelligence:
  - **Persistence**: The agent can continue to work toward a goal for an extended period despite difficulties.
  - **Plasticity**: The agent can achieve a goal from different starting points and is amenable to having the goal changed (not entirely hardwired).
  - **Diversity**: The agent has multiple ways of achieving the goal; for example, an organism may have several ways of regulating body temperature, such as sweating, panting, lowering its metabolism, sitting in the shade, and so on, and cells can build a newt kidney tubule using cell-to-cell communication or cytoskeletal bending mechanisms.
  - **Information processing**: The agent can receive, store, process, and use information needed to promote its goals; for example, information about skin rupture triggers wound healing, and regeneration of just the right amount of limb tissue in a salamander is based on information about how much was removed and how much still remains.
  - **Sensation**: The agent can receive sensory information about the environment or its own internal states that it uses to promote its goals; for example, a cockroach runs away from a light source, and cells move in response to chemical, mechanical, and electrical states of themselves and their neighboring cells.
  - **Learning**: The agent’s behavior and responses can be modified by past experience and change its behavior to achieve its goals; for example, a horse that learns to avoid an electric fence, a bee colony that learns the location of sources of nectar, or a gene regulatory network that learns to respond to a previously neutral stimulus based on its past presentation with an effective stimulus (Pavlovian conditioning (Biswas et al. 2021, 2022)).
  - **Ingenuity**: The agent can use different methods to achieve a goal, depending on the circumstances; for example, a bird may build a nest with pine needles or grass, depending on the availability of these materials.
  - **Novelty**: The agent can use new ways of achieving a goal that it has not had experience with before; for example, a bird that is trapped inside an airport builds a nest on a steel beam, or Xenobots perform kinematic self-replication when the normal frog mode of reproduction is not available to them. Novelty is different from diversity because an agent might have several ways of achieving a goal, but none of them are new.
  - **Organization**: The agent has multiple goals, including coordinated and interacting sub-goals; for example, when a gazelle flees from a cheetah, it must coordinate its breathing, heart rate, digestion, and sensory awareness to achieve the goal of survival; likewise, the process of morphogenesis must meet numerous anatomical, physiological, and metabolic constraints simultaneously.
  - **Hierarchy**: The agent has a hierarchy of goals used to coordinate goals; for example, for a gazelle fleeing from the cheetah, survival takes precedence over other goals it may have, such as feeding.
  - **Consciousness**: The agent has first-person awareness of qualitative experiences, like pain, hunger, thirst, sensations, and emotions, including valence and affect for specific states.
  - **Perception**: The agent interprets sensory information based on experience and functional awareness. Though perception involves sensation, it goes beyond sensation and involves a higher level of awareness. For example, a fly that darts from a moving object may be acting based on sensation and instinct, whereas a deer that learns to differentiate between people and other animals and flees from people is acting on perception.
  - **Mental representation**: The agent forms a mental model of the world, and means of achieving goals; for example, a baby that grabs a teething ring and sticks it in its mouth to soothe its pain, or a planarian tissue that stores a bioelectrical pattern memory of what it should build if it gets injured in the future; mental representations need not necessarily be second-order metacognitive or involve consciousness or subjective experience.
  - **Self-awareness**: The agent’s mental model includes a representation of itself and it has personal memory of its life history.
  - **Other-awareness**: The agent understands that other agents have self-awareness, thoughts, and feelings.
  - **Planning**: The agent can execute sequential, composite, and modular actions to achieve goals via sub-goals, such as acquiring materials to make a tool for feeding.
  - **Delayed gratification**: The agent can prioritize goals and delay gratification of needs, wants, or desires for longer-term goals, and is able to move temporarily away from its goal in order to recoup gains later.
  - **Problem-solving**: The agent uses its memory, perception, and representational abilities to find different possible ways of achieving goals and outcomes; the agent might, for example, be a conventional group of cells in a body, a crow that places pebbles in a pitcher to make the water level rise so as to obtain food or water (Bird and Emery 2009), or a larger-scale swarm made of living bodies, such as a group of ants that is able to manipulate a geometric shape through a maze (Dreyer et al. 2025). In each case, goals are defined with respect to different scales’ agents and their problem-spaces.
  - **Language**: The agent can use symbols to communicate information, intentions, or emotions to other agents to achieve common goals; the agent can be persuaded by linguistic communication; sophisticated language entails the use of a generative grammar.
  - **Selfhood**: Broadly conceived, a self is a term that emphasizes a first-person inner perspective consisting of valence, attention, and decision-making. Selves are owners of goals, preferences, and memories that do not belong to any of their parts—an emergent whole implemented by an interlocked triad of (1) an option space within which it operates, (2) a cognitive light cone demarcating the size of goals in that space which the system can pursue, and (3) a set of cognitive/computational processes that allow the system to navigate the space with some degree of competency. Selves live forward in time, being essentially decision-making systems that cannot be effectively described by deterministic backward-looking causal stories. Selves arise when some collection of parts all buy into the same shared story (model) of themselves and their environment (for example, when cells become coupled by gap junctions, which allows them to share physiological engrams of experiences and thus build up the same internal models/memories, and become a coherent emergent morphogenetic agent like an embryo). A defining feature of significant selves is that they do not try to observe reality “as it is” (preserving the fidelity of information about the environment’s microstates) but instead compress and coarse-grain it into memories that will allow their future instances to preserve adaptive salience, not detail (Levin 2024b). Selves interpret and reinterpret their memory engrams, just as they do with any input or signal, not being tied to how the sender (including their own past self) intended it, but doing the best they can to extract adaptive meaning in their current internal and external circumstances (this is an example of polycomputing). Selves are primarily sense-making systems, which reinforce their own reality via models of the world in which they exist.
  - **Abstract thinking**: The agent can mentally represent and manipulate abstract concepts, such as numbers, shapes, words, time, and so on.
  - **Realism**: The agent makes use of categories for distinguishing between reality and fantasy, truth and lies.
  - **Reasoning**: The agent can use deductive or inductive reasoning to make inferences, construct arguments, solve mathematical equations, draw connections between ideas, test hypotheses, and design artifacts; the agent can be persuaded by abstract arguments.
  - **Emotional maturity**: The agent has the ability to understand and manage its emotions and react appropriately to others’ emotions.
  - **Metacognition**: The agent can think about its own thinking, is critically aware of itself and its environment, and chooses its own goals, including abstract goals, such as finding meaning and purpose in existence.
  - **Morality**: The agent has moral agency; that is, the agent can act according to moral standards, use moral standards to evaluate other agents or situations, and develop and critically reflect on moral standards.
7. A key task of biology and other sciences is to understand an agent’s capacities (or its place on the spectrum of intelligence) because information about these capacities is crucial for explaining, predicting, and manipulating the agent’s behavior (Levin 2022, 2023a, c).^2^ If an agent is not capable of understanding language, then there is little point in using language to try to influence the agent’s behavior. If an agent is capable of understanding language, then using language to influence their behavior may be preferable to using brute force. There is a spectrum of persuadability corresponding to the spectrum of intelligence (Levin 2022) (see Fig. 1 in Part One). Highly intelligent agents, such as human beings, can be persuaded by language and rational arguments, while mechanical devices with no software, such as clocks, can be persuaded only by mechanistic interventions (that is, brute force). Some animals, such as dogs, horses, and mice, can be persuaded by operant conditioning. As we saw in Part One, cells connected by bioelectric networks can be persuaded by modifying their stored information. In the next section, we will discuss how understanding the behavior of bioelectric networks and developing methods for persuading (or “hacking”) them may have important applications in regenerative medicine, oncology, and other disciplines.
8. Contemporary literature on teleology and teleonomy has focused on establishing necessary and sufficient conditions for ascribing functions to traits. The approach has generated a barrage of definitions, counterexamples, and refinements. We are not interested in engaging in this dialectic because we believe that the term “function,” like many other important concepts in science and philosophy, has multiple meanings and therefore may be impossible to define by means of rigid rules.^3^ We view goal-directedness, functionality, and related ideas as family-resemblance concepts (Biletzki and Matar 2023; Wittgenstein 1953); that is, one can develop a list of characteristics associated with these concepts, but not a set of necessary and sufficient conditions for applying them. We are more interested in developing a concept of goal-directedness that has scientific and practical value than in promulgating a definition that is impervious to semantic critiques.
9. Although we have developed our approach to goal-directedness in biology to describe, explain, and predict the behavior of bioelectric networks in morphogenesis, the list of characteristics associated with goal-directedness does not depend on the method of control. Biological systems have many different methods of coordinating behavior to attain goals, including chemical signaling, physical control, behavioral communication, and symbolic language. Since our approach to goal-directedness is not tied to any of these methods, it can be applied to various systems, including ant colonies, genetic regulatory networks, bird flocks, economies, ecologies, and so on. It could also be applied to alien life forms and non-biological systems, such as artificially intelligent machines, robots, and swarms of nanobots.
10. While we are claiming that intelligence comes in degrees, we are not claiming that all matter has some degree of intelligence. To have some degree of intelligence, a system must at least have persistence and plasticity. A thermostat has such qualities, but a chunk of granite or even a diamond does not. While we want to extend the scope of mental concepts, we are not defending panpsychism.
11. Although we believe that subjective, qualitative experience emerges at some point on the spectrum of intelligence, we do not have a clear view on why or how this happens. We are not addressing the “hard problem of consciousness” (Chalmers 1997). We are only dealing with “easy” problems related to intelligence, such as agency, information processing, memory, problem-solving, cognition, and language.

## Applications: Looking Toward the Future

The validity of conceptual frameworks becomes evident from the novel research programs they facilitate and the new capabilities they enable to be visualized and pursued. We envision a number of new research directions made possible by unleashing powerful concepts from behavioral and cognitive sciences on problems ranging across biomedicine, bioengineering, ecology, evolution, and AI.

First, the recognition of problem-solving capacities in cells and tissues opens the way to research into how they perform computations that are currently intractable to our biomedicine and drug discovery efforts. For example, newt cells can build a correctly sized and shaped body despite large variability in the ploidy of cells, adapting to a new copy number of DNA and drastic changes in cell size by using novel molecular mechanisms as needed to create the same structures (Fankhauser 1945a, b). Likewise, planaria exposed to barium experience a drastic degeneration of the entire head (as barium abrogates necessary potassium exchange), but quickly regenerate a new, barium-adapted head by finding just a handful of genes among their genome whose upregulation enables them to solve this completely novel physiological stressor (Cervera et al. 2024; Emmons-Bell et al. 2019). Understanding how cells find solutions, in very large problem spaces, to novel challenges involving their own parts and the external environment could revolutionize the biomedical search for interventions to disease states. We currently have a few algorithms that can systematically identify such solutions (often relying on expensive screens), and the tools of cognitive and behavioral science are an extremely promising avenue to creating bio-inspired ways to find therapeutics.

More broadly, the many examples of plasticity of form and function with unchanged genetics (Oviedo et al. 2010; Sullivan et al. 2016) enable the same kind of powerful reprogrammability afforded by brain-based flexibility of behavior. This bodes well for coaxing new outcomes without gene therapy and intractable attempts to guess the necessary genetic changes for desired complex system-level outcomes (Lobo et al. 2014). Applications in cancer and birth defects (Chernet et al. 2015 ; Chernet and Levin 2014; Pai et al. 2015; Pai and Levin 2022) have already shown a roadmap for how the same genetically encoded hardware can be modified and repaired by signals and stimuli that exploit the agential material of life top-down (Pezzulo and Levin 2016).

Second, we have argued (Lagasse and Levin 2023; Levin 2024a) for a novel path to definitive regenerative medicine, one that avoids micromanaging symptoms with their attendant side effects and unpredictable efficacy, in favor of true solutions to injury, degeneration, aging, and cancer. This requires the discovery of storage and encoding of goal states (bioelectrical and other), which can be rewritten by therapeutics, thereby gaining buy-in from the cells (as opposed to unwanted compensatory responses). A broader version of this research program expands from directly rewriting goal states to actual training protocols in which cells’ and tissues’ basal cognition processes (Baluska et al. 2022 ; Lyon 2015; Reber and Baluska 2021) are exploited via positive and negative reinforcement and behavior shaping protocols (Abramson and Levin 2021) toward desired physiological and anatomical outcomes too complex to micromanage with current tools. Indeed, it is possible to train not only cells and tissues but also molecular pathways within cells, opening the path for drug conditioning and many applications in novel use of pharmaceuticals (Mathews et al. 2023).

Third, a broader development and use of tools from the sciences of mind impact bioengineering and the development of synthetic living machines (Ebrahimkhani and Ebisuya 2019; Ebrahimkhani and Levin 2021; Kamm and Bashir 2014; Kamm et al. 2018), with the realization that we must manage not only emergent form and function but also emergent cognition. We envision a new set of tools for programming biobots with stimuli and experiences, not only synthetic biology circuits. Beyond the utility of these machines for us, the realization of degrees of mind in unconventional embodiments (Rouleau and Levin 2023, 2024) forces sharper questions of ethics and what we owe the agents that we create. No longer is it sufficient to compare organoids to human brains, or to assume that lack of conventional sense organs and motility in an in vitro construct means that it’s not “embodied” (not running perception–action goal-directed loops in physiological and other spaces that are hard for us to see) (Fields and Levin 2022). Moving away from human cognition as a standard in ethical models toward a realization of diverse embodiments gives rise to a robust research program to expand ethical frameworks built on unsustainable ancient binary categories and update them in light of the recent science of diverse intelligence.

An important frontier for combating the mind-blindness and teleophobia that have held back progress is the development of conceptual and practical tools for communication and collaboration with unconventional and non-linguistic systems. The future use of AI and other tools as a kind of translator module between radically different types of minds will enable a new frontier of more ethical *synthbiosis*, from bioengineered life to entire ecosystems. Especially critical is the fact that this set of frameworks enables a much more natural expansion of ethical, legal, and social structures to encompass the forthcoming cyborgs, hybrots, and other composite beings that cannot readily be assigned to the human (or life) versus machine categories. The forthcoming variety of human and nonhuman minds, far broader than Darwin’s original “endless forms most beautiful” (Clawson and Levin 2022), requires a more sophisticated framework and cannot be dealt with by current binary categorizations.

## Objections and Replies

Before concluding our essay, we would like to address a few key objections to provide further support for our view and help the reader better understand it.

### First Objection:

You are implying that scientists and philosophers should abandon current approaches to goal-directedness, which seem to do a pretty good job of explaining some important cases, such as homeostatic regulation and the evolution of biological adaptations. Why throw out the baby with the bathwater?

### Reply:

As we stressed in the second section of this article, we are not arguing that these other approaches should be rejected, but we are arguing they cannot adequately explain, and more importantly, extend, the experimental approaches described in the section on experimental results in Part One of this article and therefore a new approach, which more effectively drives research into the competencies of unconventional embodiments of mind (with its many practical applications), is needed. Current approaches could be used, depending on the type of goal-directedness that scientists are trying to understand. Some types of low-level goal-directedness, such as homeostasis, might be best explained in terms of causal roles and relationships with no mention of agency or intelligence. Biological adaptations, in many cases, could be explained in terms of the operation of natural selection over time and nothing more. We see no need to interfere with the use of explanatory frameworks that yield fruitful results. Since we accept multiple approaches to goal-directedness, our view could be characterized as pluralistic and inclusive, even though it represents a conceptual shift toward mentalism. We argue that, on the spectrum of agency, the goal should be to empirically determine the optimal position for a given system—not intentionally skew low (Morgan’s Canon (Morgan 1903) nor high (animism) but use experiments to determine which discipline (ranging across physics, cybernetics, behavioral science, psychoanalysis, and others) provides the most payoff in terms of prediction, control, and synthetic discovery of new vistas of research.

### Second Objection:

Your approach is incompatible with scientific materialism because it involves mentalism, which is too high a price to pay for explaining experimental results. Scientists and philosophers have worked hard since the 1950s to show how teleonomic language in biology provides materialistic explanations of goal-directedness, and your approach would undo all that.

### Reply:

As we have argued in Part One of this article, we think that our approach is compatible with scientific materialism, because we are not assuming the existence of metaphysically irreducible minds. We hold that mind, intelligence, agency, and related terms are ultimately compatible with the objects, processes, and properties belonging to physics and chemistry (Levin 2022; Levin and Dennett 2020). While cognitive scientists, neuroscientists, and philosophers have made tremendous progress toward understanding how the human mind functions over the last 50 years, their achievements are still decades away. We readily acknowledge that the mind–body problem remains one of science’s great mysteries (Chalmers 1997, 2010), but we argue that the way to make progress on this and other problems is to move away from the implicit dualism of artificial sharp boundaries between canonical mindful entities and their more primitive origins and instead develop principled research programs that seek to understand the scaling and transformation of diverse cognitive capacities on evolutionary and ontogenic timescales.

### **Third Objection**:

Using the word “intelligence” and other cognitive terms outside of their familiar context of brainy animals immediately raises questions: might not these terms be misused? Are not morphogenetic systems simply following the rules of chemistry—why anthropomorphize them?

### **Reply**: 

First, in the modern age, we must accept that *all* cognitive systems—ourselves included—exhibit chemistry, not magic, when one drills down to examine the lower levels. Thus, there simply is no special human category that one can correctly anthropomorphize as somehow being beyond the laws of physics at its base. We argue that this word—“intelligence”—is in some ways an anachronism that needs to be reenvisioned in favor of an empirically grounded view, updated with the latest findings in causal information theory (Albantakis et al. 2017; Hoel and Levin 2020; Hoel 2017, 2018; Hoel et al. 2013, 2016; Moore et al. 2018), in which it is perfectly possible (in fact, unavoidable) for a system to both, be subject to chemistry, and also to possess additional levels of description and control whose recognition affords novel benefits. We offer two points in clarifying our use of this terminology (developed in detail in Levin 2022). An uncontroversial aspect of our view is that claims of intelligence (and other cognitive terms), like all others, must be based on rigorous experiment, supported or ruled out by the degree of objective benefit that a given framing affords in terms of a) prediction and control, and b) future discovery (and new research programs) it suggests. The latter is most significant because almost any paradigm can be rescued by enough epicycles; indeed, after one has discovered a new effect or reached a new capability, it is easy to drill down to the chemistry and, looking backward, claim that there is no intelligence here because it mechanically follows the laws of physics. The same is true for any act of a complex human brain–body system—if one insists on a view from the level of particles, it will always be there. The key question is: does that level of perspective provide the most interesting platform from which to make the next discovery or develop the most effective control policy? The emphasis should be on novel capabilities, and new research programs facilitated (or suppressed) by a given perspective. Thus, we propose that attempts to mine the rich toolbox of behavioral science to understand and exploit capabilities of morphogenetic systems will continue to pay off in many (but no doubt, not all) cases. We have fleshed out the prior gains facilitated by this view, as well as the promises for regenerative medicine, elsewhere (Lagasse and Levin 2023; Mathews et al. 2023). The less conventional, and sometimes uncomfortable, aspect of our position is that the empirical utility of framings needs to be applied fearlessly and followed wherever it may lead: its empirical consequences must be taken seriously even when they contradict long-cherished a priori commitments to how non-intelligent a non-brainy system must be. In other words, if a specific framing, which uses tools usually reserved for brains, results in fruitful new research programs on bacterial biofilms (Martinez-Corral et al. 2019; Prindle et al. 2015; Yang et al. 2020), plant roots (Bassel 2018; Calvo et al. 2016; Ciszak et al. 2016; Davis et al. 2023; Johnston and Bassel 2018; Topham et al. 2017; Yokawa et al. 2018), the training of gene-regulatory networks (Biswas et al.^2021^ , ^2022^; Csermely et al. 2020; Gyurkó et al. 2013), or developmental/regenerative biology (Levin 2021), then the scientific approach requires that we consider those systems to be bona fide subjects of that corner of the natural world that is supposed to be described by the behavioral science of a spectrum of minds.

### Fourth Objection

Aren’t you mistaking emergent complexity and unpredictability for intelligence? Is there really a research program here?

### Reply

Explicitly, we hold that the reason to attribute intelligence to a process such as morphogenesis is *not* because it looks complex, unpredictable, agential, or anything else. The only reason to attribute a specific degree of intelligence (and claim specific capabilities as listed above) is to do perturbative experiments that reveal them. These competencies cannot be inferred from strictly observational data, and complexity alone is insufficient—different types of problem-solving capacities need to be explicitly demonstrated in functional experiments. Likewise, they cannot be ruled out based on philosophical commitments or classical categories and colloquial language use but must be argued against based on experimental evidence. The research program we propose is rigorously focused on empirically testing, not historically assuming, the boundaries between disciplines that have suppressed new discoveries by the gate-keeping threat of “category errors”. The categories must evolve with the science, not be maintained for historical reasons. Just as people are using tools of cognitive science to rigorously test the capacities of swarm intelligence (Couzin 2007, 2009; Dreyer et al. 2025) in collectives of animals (going up a level from conventional individuals), we emphasize the utility of testing these tools in novel substrates and at novel scales (Baluska et al.^ 2022^; Baluska and Reber 2021 ; Calvo et al. 2017, 2020; Deisboeck and Couzin 2009; Levin 2023c; Mori and Koaze 2013; Reber and Baluska 2021 2021; Reid et al. 2013; Saigusa et al. 2008; Trewavas 2016; Vallverdu et al. 2018; Zakirov et al. 2021). In all of these cases, an operational and pragmatic view of cognitive terms suggests that when their application leads to progress and new discoveries, they are legitimately applicable.

### Fifth Objection

Isn’t it odd to expect cognition in non-brainy substrates—why would it be there?

### Reply

On the contrary, taking evolution seriously requires that there be ancestral functions leading up to the development of the most obvious features of brains. Indeed, it is now known (Fields et al. 2020; Keijzer et al. 2013) that all of the mechanisms used by the brain to carry out its amazing functions—ion channels, electrical synapses, neurotransmitters—are evolutionarily ancient and are already doing brain-like signaling in bacterial biofilms. Not only the mechanisms, but many of the algorithms around information processing, active inference, and so on, are widely conserved beyond brains (Friston et al. 2015; Kuchling et al. 2020; Pezzulo and Levin 2015). Thus, if either mechanistic composition or functional behavior is taken as the kind of things that provide evidence for cognition, there should be no surprise to find it in the living material that was speed-optimized and augmented to become the central nervous system.

## Conclusion

Returning to where we began in Part One, since Aristotle’s time, scientists have been trying to understand purposiveness in the living world. Attempts to banish teleological concepts from the life sciences have proven to be largely unsuccessful because (a) life exhibits myriad forms of goal-directedness that cannot be easily explained in causal-mechanical terms and (b) teleological concepts, such as goal, purpose, and function, are useful in hypothesis formation, model building, and experimental design in biology. In response to this predicament, scientists and philosophers have proposed theories of biological teleology that avoid the use of methodologically “dubious” mentalistic concepts and explain goal-directedness by referring to properties, processes, and objects that seem to be compatible with a mechanistic worldview, such as genetic program, causal role, adaptation, and field. While these non-mentalistic approaches to teleology can account for many phenomena in biology and other domains, they are not capable of adequately explaining ground-breaking experimental results in morphogenesis, synthetic biology, and other fields, as described in this article, which involve the manipulation of cells and tissues connected by bioelectric networks. In this article, we have defended a mentalistic approach to goal-directedness to account for these and other experimental results, but the view also applies to other biological systems that exhibit complex, goal-directed behaviors, such as ant colonies, economies, and genetic regulatory networks. Our view is motivated not by the desire to prove any philosophical points but by the practical necessity of developing concepts that can fruitfully guide inquiry in these fields of research and suggest testable hypotheses, explanatorily robust models, and theories.

Based on the arguments and conceptual clarifications we have made in this two-part article, and examples of real research programs that are not only consistent with, but actually driven by, the ideas we have defended, it is clear that a continuous, pluralistic, and pragmatic approach to agency, mind, and cognition beyond the brain is possible. The field of diverse intelligence offers numerous opportunities to move beyond the increasingly limiting teleophobia and animism of the past, toward developing principled theories that port tools and concepts across the increasingly blurring boundaries of disciplines that were established centuries ago. Truly transdisciplinary work, spanning science and philosophy, can now be done to positively impact clarity about the status of embodied minds and the biomedicine, engineering, and other practical advances that await our ability to optimally interact with intelligence in unfamiliar guises.

## Footnotes

1 This is proposal open to revision. We may have omitted some characteristics that should be on the list. See Gardner ( 2011) for some criteria for human intelligence.

2 Lee and McShea ( 2020) have developed a methodology for measuring goal-directedness. While we think that measurement can play a key role in advancing our understanding of goal-directedness, we are not sure whether it is possible to accurately, precisely, and consistently measure goal-directedness when a system has more than a rudimentary level of intelligence, due to the complexity of the system and the number of different metrics that might apply. Rather than trying to assign a numeric value to the goal-directedness of a system, it may be more fruitful to describe the system’s capacities and place it on a relative scale. That is, to measure goal-directedness qualitatively rather than quantitatively.

3 “Life” may also resist definition by means of rigid rules (Gómez-Márquez 2021).
